# Postural motion perception during vestibular stimulation depends on the motion perception threshold in persistent postural-perceptual dizziness

**DOI:** 10.1007/s00415-024-12415-z

**Published:** 2024-05-15

**Authors:** Christoph Helmchen, Smila-Karlotta Blüm, Renana Storm, Janina Krause, Andreas Sprenger

**Affiliations:** 1 Department of Neurology, University Hospital Schleswig-Holstein, University of Lübeck, Campus Lübeck, Ratzeburger Allee 160, 23538 Lübeck, Germany; 2 Center of Brain, Behavior and Metabolism (CBBM), University of Lübeck, Ratzeburger Allee 160, 23562 Lübeck, Germany; 3 Institute of Psychology II, University Lübeck, Ratzeburger Allee 160, 23562 Lübeck, Germany

**Keywords:** Persistent postural-perceptual dizziness, PPPD, Postural motion perception, Sensory thresholds, Vestibular, Functional disorder

## Abstract

Patients with persistent postural-perceptual dizziness (PPPD) perceive postural instability larger than the observed sway. It is unknown whether the concept of postural misperception prevails during vestibular stimulation and whether it may account for the unsteadiness patients complain during body movements. We tested the hypothesis of an abnormal sensory-perceptual scaling mechanism in PPPD by recording objective, perceived, and the reproduced postural sway under various standing conditions, modulating visual and proprioceptive input, by binaural galvanic vestibular stimulation (GVS). We related postural sway speed to individual vestibular motion perceptional thresholds and disease-related PPPD questionnaires in 32 patients and 28 age-matched healthy control subjects (HC). All participants showed normal vestibular function tests on quantitative testing at the time of enrollment. The perception threshold of GVS was lower in patients. Compared to HC, patients showed and perceived larger sway on the firm platform. With GVS, posturo-perceptual ratios did not show group differences. The ratio of reproduced to real postural sway showed no group differences indicating normal postural sway perception during vestibular stimulation. Noticeably, only in patients, reproduced postural instability became larger with lower individual thresholds of vestibular motion detection. We conclude that posturo-perceptual (metacognitive) scaling of postural control seems to be largely preserved in PPPD during GVS. Vestibular stimulation does not destabilize patients more than HC, even in challenging postural conditions. Low individual thresholds of vestibular motion perception seem to facilitate instability and postural misperception on solid grounds. This conclusion is important for an effective physical therapy with vestibular exercises in PPPD.

## Introduction

Persistent postural-perceptual dizziness (PPPD) is a frequent and chronic (> 3 months) disorder of perceived unsteadiness in the absence of peripheral sensory abnormalities [1]. The Bárány Society established its diagnostic criteria in 2017 [2]. Symptoms are often exacerbated by upright standing posture, active or passive motion or exposure to visual stimuli. Many patients experience a previous event destabilizing posture, e.g., episodic (vestibular neuritis) or recurrent vestibular disorders (benign positional paroxysmal vertigo, BPPV [3], syncope, or prolonged physiological multisensory stimulation, e.g Mal de debarquement syndrome) [4]. As PPPD severity it is not the magnitude of previous or still persistent vestibular dysfunction [5] a maladaptation of perceived postural control in response to several related mechanisms is discussed: (i) predictive processing of sensory inputs by abnormal bottom-up central processing of self and external motion signals, (ii) a misprediction of the sensory consequence of one ‘s own movements (efference copy), and (iii) alterations in motion perception that influences top-down postural control [6–8]. A mismatch between ‘bottom-up’ (vestibular/proprioceptive sensory) inputs and maladaptive signals from ‘top-down’ attentional control systems has been suggested to determine perceived postural imbalance [8]. One trigger may come from altered thresholds of sensory (visual, vestibular, proprioceptive) motion perception, which have been studied in response to galvanic vestibular stimulation [9], to passive rotatory turntable [10, 11] or rotatory roll tilt or translational z-vestibular motion [12].

PPPD patients preferably seem to rely on visual inputs for balance control compared to other sensory, i.e., vestibular and somatosensory inputs [8, 13]. Sensitivity to moving visual stimuli often becomes annoying in PPPD. This may be related to an abnormal sensitivity in the visual cortex, e.g., in response to virtual reality moving scenes or in the rod-and-disk test [14, 15]. This visual dependence resembles functional neural reorganization in patients who suffered from unilateral vestibulopathy with subsequently developing abnormal visual impact on vestibular perception during the course of the disease [16, 17]. In PPPD, we recently demonstrated poorer visual motion detection in PPPD [11]. Vestibular motion perception thresholds are lower during binaural galvanic vestibular stimulation but not during rotatory vestibular stimulation around the earth-vertical axis [11, 12].

Altered thresholds of sensory perception might lead to subjective postural unsteadiness which in turn could facilitate inappropriate postural motor responses to sensory feedback and inappropriate adjustments [18]. However, only a few studies have examined postural control in PPPD under different sensory conditions yet. In phobic postural vertigo (PPV), a related (predecessor) disorder or a subtype of PPPD [2], large-field visual motion stimulation in the roll plane elicited smaller lateral body sway in PPV patients than in healthy subjects which has been related to a lower sensory threshold at which they initiate a compensatory body sway opposite to the direction of the perceived body deviation [19]. Abnormal postural sway during normal stance on a firm platform paradoxically improved with more difficult balance tasks (tandem stance) [20]. Functional disorders have been proposed to be associated with “excessive” conscious monitoring of one’s own movement [21]. Evidence for an exaggerated attentional focus on postural adjustments [22] came from studies showing that cognitive distraction in dual-task conditions improved postural sway of PPPD patients [9, 23]. Using posturography, PPV patients showed a postural behavior in an easy balance condition (normal stance with visual control) that is usually found in healthy subjects under more demanding postural conditions (tandem stance, eyes closed) when they focus attention on balance maintenance [13, 23, 24]. These changes are compatible with higher levels of anti-gravity muscle activity and co-contraction during focused attention on one’s own postural stability [23]. Impairment of postural control in PPV was, therefore, suspected to arise from a lowered sensory feedback threshold of the balance control system [25]. This is crucial as postural control in PPPD is shifted from a feed-forward (governed by motor commands placing the subjects into the desired position) to a largely feed-backward driven control mode (e.g., sensory signals) [23, 25].

Using the EquiTest® Sensory Organization Test (SOT), PPPD patients revealed abnormal sway scores in all domains, not only on deprivation of multiple sensory inputs but also of one sensory input only [26]. Visual dependence was reflected by higher Visual Vertigo Analog Scores and a greater anterior–posterior sway of PPPD patients [27] but postural imbalance was not different compared to non-PPPD dizzy patients [15]. Additional lines of evidence for a stronger visual dependence of postural control in PPPD came recently from foam posturography showing a higher Romberg’s ratio in PPPD patients [28].

Postural control has been studied in PPPD with various conditions of selective withdrawal of sensory input stabilizing posture [26–28] but do PPPD patients use vestibular signals properly for their postural control during vestibular stimulation? Interestingly, galvanic vestibular stimulation with intensities eliciting postural sway and egomotion perception in PPPD patients elicited higher sway velocity under visual suppression compared to healthy subjects which reversed under more difficult balance conditions with somatosensory deprivation (foam) [9].

The patients’ perception of their own postural balance was studied very recently [29], irrespective of potentially altered sensory thresholds. There was a strong mismatch between observed and perceived postural instability: PPPD patients perceived their small postural sway much stronger compared to patients with vestibulopathy with a larger objective sway. When asked to reproduce the perceived sway of the baseline recording in darkness patients showed a much higher postural sway with the eyes open reflecting severe postural misperception, i.e., recorded and reproduced sway did not match indicating an abnormal scaling mechanism. Interestingly, this misperception reversed in a metacognitive intervention by providing a feedback of the recorded sway thereby reducing the magnitude of postural sway perception and its reproduced sway.

It is unknown whether this postural misperception in PPPD also occurs during vestibular stimulation (e.g., locomotion) and whether it accounts for the egomotion and imbalance patients complain during body movements in the absence of vestibular failure. We tested the hypothesis of an abnormal sensory-perceptual scaling mechanism in PPPD by recording objective, perceived and the reproduced postural sway on a Kistler platform under various conditions modulating visual and proprioceptive input (eyes open/closed, firm platform vs. foam) by binaural suprathreshold galvanic vestibular stimulation [9]. We related postural sway velocity to the magnitude of perceived motion and the individual vestibular motion perceptional thresholds as well as disease-related questionnaires of PPPD patients and age-matched healthy control subjects (HC). To exclude confounds by persisting vestibular disease, all participants showed normal vestibular function tests on quantitative testing at the time of enrollment.

## Methods

### Participants

Thirty-two PPPD patients and 28 age-matched healthy control subjects (HC) were included in this study. All patients met the diagnostic PPPD criteria of the Bárány Society [2]. Age-matched healthy subjects had no history of vertigo, dizziness, migraine or other types of balance disorders. Most of the subjects participated in a related study investigating their visual and vestibular motion perception thresholds [11]. Demographics and patient characteristics are summarized in Table 1, including questionnaires addressing motion sickness susceptibility, dizziness intensity, impact on daily life, level of anxiety, depression, and personality features. We used the validated Niigata Questionnaire on PPPD [30], the Motion Sickness Susceptibility Questionnaire (MSSQ) [31]; the Neuroticism and Extraversion scores of the 5-Factor Inventory Personality Questionnaire (NEO-FFI )[32], anxiety and depression scores of the Hospital Anxiety and Depression scale (HADS) [33, 34], a questionnaire distinguishing PPPD symptoms in the context of egomotion, rest, head motion, and visual motion, i.e., the Athens-Lübeck-Questionnaire on PPPD (ALQ) [35], and the State and Visual Analog Values of the EQ-5D-3L [36]. In short, PPPD participants revealed higher values of symptom exacerbation by visual and egomotion (Niigata questionnaire for PPPD, MSSQ, ALQ), for neuroticism (NEO-FFI), and of anxiety and depression (HADS); and self-assessment for daily life quality (EQ-5D-3L). None of the PPPD patients was on medication affecting CNS at the time of recording, specifically participants did not take any medication affecting cognition, pain or mood.

**Table 1:** Demographics and clinical scores of participants

	PPPD (mean ± STD)	Healthy subjects (mean ± STD)	Statistical difference *p*
Number	32	28	n.s.
Age	43.7 ± 11.4	43.4 ± 12.6	n.s. (*p* > 0.9)
Gender (f:m)	21:11	16:12	n.s.
Disease duration (months)	33.6 ± 25.0	–	–
Niigata score	27.4 ± 13.4	1.9 ± 3.1	< 0.001
MSSQ	13.3 ± 11.5	8.1 ± 8.3	0.049
Neuroticism (NEO-FFI)	22.2 ± 4.1	20.0 ± 5.2	0.070
Extraversion NEO-FFI)	25.3 ± 4.1	25.4 ± 5.1	0.878
ALQ	16.1 ± 7.3	0.7 ± 1.7	< 0.001
HADS-A	8.3 ± 5.8	3.9 ± 2.6	< 0.001
HADS-D	5.8 ± 3.7	2.2 ± 2.2	< 0.001
EQ VAS	64 ± 18	89 ± 11	< 0.001

*MSSQ* Motion Sickness Susceptibility Questionnaire, *NEO-FFI* Neuroticism and Extraversion scores of the 5-Factor Inventory Personality, Questionnaire, *HADS* Hospital Anxiety and Depression scale, *ALQ* Athens-Lübeck-Questionnaire, *EQ VAS* visual analog scale of the EQ-5D-3L

PPPD patients were recruited from the University Centre for Vertigo and Balance Disorders. Only patients with a disease duration of > 3 months were included. All participants underwent a second clinical neurological and neuro-otological examination at the day of behavioral recordings. None of the participants had clinical signs of a persistent vestibular hypofunction, positional nystagmus, cerebellar dysfunction, and all of them had normal visual acuity. Exclusion criteria included persistent vestibular failure (gain < 0.7 of horizontal VOR gain), dementia, major depression, personality disorders, polyneuropathy, sedative drugs, consumption of alcohol, and the inability to stand without assistance. None of the patients had abnormal vestibular functions on clinical and quantitative recordings (quantitative head impulse test, caloric irrigation, vestibular evoked myogenic potentials, subjective visual vertical) at the time of enrollment. Previous vestibular episodes included benign paroxysmal vertigo, unilateral vestibulopathy, exposure to moving platforms but clinical and vestibular function tests demonstrated complete restitution (VOR gain > 0.7) before recruitment in this study.

The study protocol was conducted in accordance with the Declaration of Helsinki and its later amendments and approved by the local Ethics Committee of the University of Lübeck (AZ 17-036, AZ 21-098) and written informed consent was obtained from all participants.

#### Electrophysiological and psychophysical recordings of vestibular function

All standardized vestibular tests showed data within normal limits and no group differences. Vestibular function of participants was examined by video-oculography with caloric irrigation [bithermal cold (27°) and warm (44°) caloric irrigation] and quantitative head impulse testing (qHIT). Eye and head movements were recorded by the EyeSeeCam® HIT System (Autronics, Hamburg, Germany) at a sampling rate of 220 Hz. Quantitative HIT was delivered by passive head impulses (HIT) with rapid small amplitude (10–15°) horizontal head rotations (3000–4500°/s^2^) while the participant was sitting on a chair fixating a red LED at a distance of 100 cm. For further details, see [9, 37–41]. Psychophysical perception of the visual vertical was assessed with the head fixed on a chin rest by the subject’s adjustment of a bar to the perceived visual vertical without any spatial orientation clues in a dotted half-spherical dome, which is stationary or dynamic (moving visual background) around the line of sight [42]. The normal range of SVV was defined as deviation of < 2.5°.

#### Posturography

We used a Kistler force platform (Model 9260AA6, Kistler Instrumente AG, Winterthur, Switzerland; 50 cm width, 60 cm length) equipped with piezo-electric 3-component force sensors for recording postural changes during the above mentioned experimental conditions in a similar way as described elsewhere [43, 44]. The platform recorded torques and sheer forces with six degrees of freedom using force transducers with an accuracy better than 0.5 N. The displacement of the center of pressure (CoP) in the medial–lateral (ML) and the anterior–posterior (AP) directions were recorded and the sum vector calculated using Matlab® (R2022b, The Mathworks, Natick/MA). Results are given as the median postural sway speed (PSS, in cm/s), calculated from the anterior–posterior (AP) and medio-lateral (ML) movements:

$${\text{PSS}} = {\text{median}}\left( {\sqrt {\left( {AP_{i} - AP_{i - 1} } \right)^{2} + \left( {ML_{i} - ML_{i - 1} } \right)^{2} } *SamplingRate} \right).$$

Postural sway was recorded in intervals of 20-s duration for off-line analysis (sampling frequency 250 Hz) [45, 46]. PSS has been shown as a robust, discriminative, and reliable factor of recording postural balance [47, 48].

#### Galvanic vestibular stimulation (GVS)

The current stimulator (DS5 model, Digitimer Ltd., U.K.) delivered bilateral mastoid galvanic stimulation with skin contact electrodes provided by EasyCap GmbH (Herrsching/Germany). This stimulator has also been used and approved in other centers and studies, e.g., [49, 50]. The stimulation site was pre-treated with local anesthetics prior the experiment (Anesderm® lotion, Pierre Fabre Dermo-Kosmetik GmbH, Freiburg/Germany) to minimize potential nociceptive stimulation of higher GVS. The skin surface was cleaned again and dried before contact electrodes with commercial contact paste were attached above the mastoid bilaterally.

Individual sensory (vestibular) thresholds were obtained by applying 10 s 1 Hz alternating stimulation, i.e., low-frequency alternating current which passed between the two mastoid electrodes [11]. The ramp stimulus profile hampered sharp transients at stimulus onset and offset (ramp onset and offset of 100-ms duration) with a stimulation plateau of 300 ms leading to perceived head and body tilt**.** Threshold testing started with an above-threshold current (usually 1 mA). Subsequently, the stimulus intensity decreased gradually in steps of 0.05 mA until the subject reported no vestibular sensations anymore. Then the procedure started again from a low threshold (0.10 mA) gradually increasing in steps of 0.05 mA until the subject reported vestibular sensations again, i.e., a perception of body motion. The threshold was verified by varying the stimulation intensity until a stable threshold was found. All subjects indicated a medio-lateral motion direction. Previous studies have shown that thresholds obtained using perceptional responses were not different from those obtained by GVS induced quantitatively analyzed body motion [51].

The following 3 stimulations were used: (i) no current (noGVS), (ii) a high intensity current (1.5 mA above the perceived threshold, highGVS), i.e., a stimulus reliably eliciting vestibular motion perception that allows to compare equivalent perceptions of vestibular motion, and (iii) a fixed above-threshold stimulus intensity, i.e., applying the same physical stimulus for all subjects (1.3 mA, independent of individual motion perception threshold, fixGVS). The fixGVS stimulus was lower (about 0.4–0.5 mA) compared to highGVS.

Each GVS stimulus was examined once in each experimental condition (eyes open vs. closed, firm platform vs. foam, 3 × GVS), with the eyes open during fixation of a gaze straight ahead target (computer screen at 60 cm) and with the eyes closed in each experimental condition resulting in 18 recording sessions (20 s each).

#### Experimental conditions

**Fig. 1:**
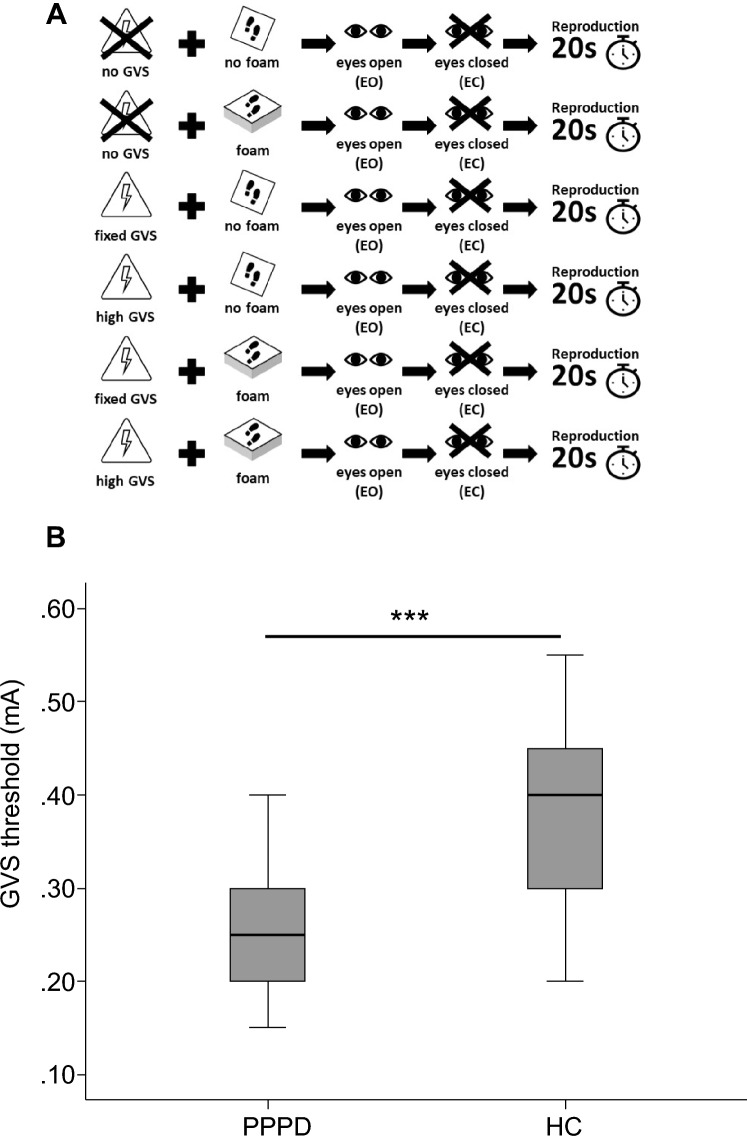
Experimental design and thresholds of vestibular motion perception. **A** The experimental design tested various combinations of the factors STIMULUS [noGVS, fixed (1.3 mA) and high GVS intensity (1.5 mA above the individual threshold of egomotion perception)] of binaural galvanic vestibular stimulation (GVS), SENSORY (firm platform, foam) and VISION (eyes open vs. closed) resulting in a 2–2-3-factorial design with a between factor GROUP. Due to technical reasons, we did not randomize the sequence of the experimental conditions. Rating of perceived egomotion was indicated on a visual analog scale on a computer screen in gaze straight ahead immediately after each experimental condition (trial) by moving a joy stick. After each condition with eyes closed, patients were requested to reproduce the perceived sway of the previous stimulation period with the eyes open for 20 s. **B** Mean thresholds of perceived egomotion are shown for GVS in PPPD and HC

Posturography was recorded in the upright standing position with the hands hanging next to the trunk for 20 s. At baseline, subjects were asked to stand on the firm platform with feet (shoes) parallel to each other. We tested the influence of the 3 GVS stimuli (noGVS, highGVS, fixGVS) on postural sway speed (PSS) under different visual (eyes open vs. closed; EO/EC) and proprioceptive feedback conditions (firm platform vs. foam), for details see [9, 46, 52] (Fig. 1). For technical reasons, we did not randomize the sequence of the experimental conditions. Participants were asked to fixate a target 60 cm in front of the participants’ forehead. We used a slab of foam rubber (50 width, 60 cm length, height 10 cm, compression hardness: 3.3 kPa, volumetric weight: 40 kg/m^3^) for testing balance control under attenuated proprioceptive feedback. After each 20 s trial subjects were asked to rate the magnitude of perceived sway on a visual analog scale (0–100, 0 = no motion perception at all, 100 = unsteadiness requiring assistance) displayed on a computer screen (at 60 cm in gaze straight ahead position) by using a joy stick.

After each trial with the eyes closed, patients and healthy subjects were asked to reproduce the postural sway with the eyes open for 20 s on the Kistler platform that they perceived during the last preceding GVS trial on the firm platform or on foam, i.e., they moved their body on the Kistler platform as they remembered they were swaying with the eyes closed. This reproduced sway was performed with the eyes open, for better comparison with a related study [29]. Sway behavior of both groups were compared in identical experimental conditions in our study. This was not the case in HC in a related study in which postural sway of patients on the firm platform was compared with sway of HC on foam to elicit some postural motion perception and in which reproduction was performed with the eyes open [29]. Postural perception was calculated by the ratio of reproduced/real (observed) sway speed, i.e., perceptual-postural ratio (PPR), i.e., a PPR > 1 indicated that the perceived and replicated sway exceeds the real (measured) sway.

### Statistical analyses

Power calculation prior the study predicted a necessary group size of at least 23 subjects per group [53]. We used a four (2 × 2 × 3 × 2)-factorial study design with the factors: VISION (eyes open, eyes closed), SOMATOSENSORY (firm platform vs. foam), STIMULATION (noGVS vs. highGVS vs fixedGVS) and GROUP. GVS, VISION, and SOMATOSENSORY were taken as within-subject factors (repetitive runs) and GROUP (patient vs healthy controls) as between-subjects factor using multi-factorial ANOVA. Statistical analyses were performed with SPSS (22.0.0.2; IBM Corp., Somer NY). Statistical comparisons were performed non-parametric unless stated otherwise. In some comparisons sphericity requirement was violated. Rank transformed data were used in case of ANOVAs with more than one factor. Therefore, we report *F*-values with Greenhouse–Geisser correction but report degrees of freedom (*df*) uncorrected to show the factorial analysis design. Significance levels of post hoc tests were Bonferroni corrected for multiple testing. Statistical differences were regarded as significant for values *p* < 0.05. Correlation analyses were performed using Spearman-Rho coefficient unless otherwise stated. Results are presented in box plots (with median, upper, and lower quartiles, e.g., 75 and 25% percentiles) for the healthy and PPPD participants. Correlation analyses were performed using Spearman-Rho coefficient unless otherwise stated.

## Results

Diagnostic workup of vestibular function (video-oculography; i.e. quantitative video head impulse test of the horizontal angular vestibulo-ocular reflex, caloric irrigation, subjective visual vertical using stationary and dynamic conditions, ocular vestibular evoked myogenic potentials) revealed normal vestibular function at the time of participants’ inclusion into the study.

### Psychophysics

The perception threshold of GVS was significant lower in PPPD patients compared to controls (*Z* = – 3.551, *p* = 0.001, Fig. 1). Participants reported no pain during GVS.

Postural sway speed (PSS in mm/s) and rating of perceived sway for each experimental condition were analyzed separately but presented together above each other in one figure for each experimental condition.

### Postural data (objective sway)

Generally, there were main effects of VISION (Eyes open vs. closed, EO EC; *F*(1,56) = 209.334, *p* < 0.001), STIMULATION (*F*(2,55) = 70.11, *p* < 0.001), SOMATOSENSORY (firm platform vs. foam; *F*(1,56) = 130.09, *p* < 0.001), and GROUP (*F*(1,56) = 8.24, *p* = 0.006). Of note, there were significant interactions of GROUPxVISION (*F*(1,56) = 4.03, *p* = 0.049), GROUPxSOMATOSENSORY xSTIMULATION (*F(*2,55) = 5.86, *p* = 0.011).

**Fig. 2:**
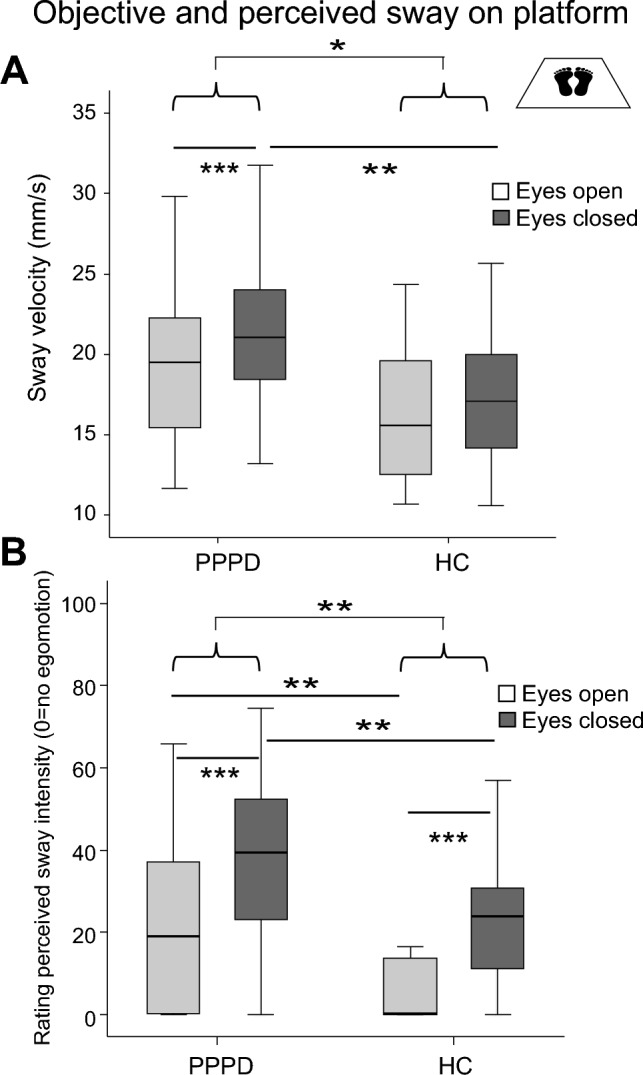
Objective and perceived sway on the firm platform. Postural sway velocity (mm/s) on the firm platform (**A**) and perceived sway intensity (**B**) on a visual analog scale (0–100, 0 = no egomotion) are shown with the eyes open and closed. PPPD patients showed a larger sway speed and perceived larger egomotion. *p = 0.05; **p = 0.01; ***p = 0.001

On the firm platform (Fig. 2), there were main effects of VISION (Eyes open vs. closed, EO/EC; *F*(1,58) = 19.73, *p* < 0.001) and GROUP (*F*(1,58) = 5.55, *p* = 0.022) but no interaction VISIONxGROUP (F(1,58) = 3.58, p = 0.064). PSS of patients with the eyes closed was larger compared to HC (*F*(1,58) = 7.87, *p* = 0.007), but not with the eyes open (*F*(1,58) = 3.23, *p* = 0.077) (Fig. 2A). Within group comparison revealed larger PSS of patients (but not HC, P = 0.086) with the eyes closed compared to open eyes (*F*(1,58) = 21.486, *p* = 0.001). *Rating of perceived sway on the firm platform:* There were main effects of VISION (*F*(1,57) = 51.775, *p* = 0.001) and GROUP (*p* = 0.006) but no interaction for VISIONxGROUP (*p* = 0.566). Patients rated perceived sway larger (29.61 ± 3.06) than HC (16.62 ± 3.34; *p* = 0.006) (Fig. 2B), both with the eyes closed (p = 0.006) and the eyes open (*p* = 0.02).

**Fig. 3:**
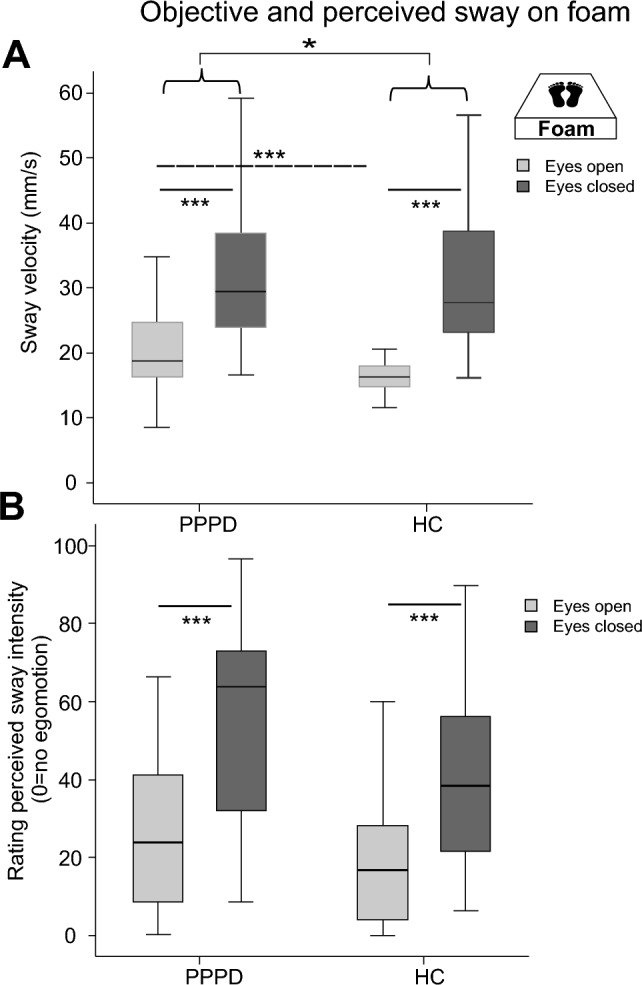
Objective and perceived sway on foam. Postural sway velocity (mm/s) on foam (**A**) and perceived sway intensity (**B**) on a visual analog scale (0–100, 0 = no egomotion) are shown with the eyes open and closed. PPPD patients showed a larger sway with visual control but did not perceive this sway larger compared to HC. *p = 0.05; ***p = 0.001

On foam (Fig. 3), there were main effects of VISION (*F*(1,58) = 100.98, *p* = 0.001) and GROUP (*p* = 0.019) and a significant interaction VISIONxGROUP (*F*(1,58) = 4.91, *p* = 0.031). Post hoc t-test showed larger PSS during eye closure in both groups (*p* < 0.001). In contrast to eye closure (*p* = 0.641), PSS of patients with the eyes open was larger compared to HC (*F*(1,58) = 9.79, *p* = 0.003, Fig. 3A). *Rating of perceived sway on foam:* There was a main effect of VISION (*F*(1,54) = 79.99, *p* < 0.001) but not of GROUP (p = 0.106) and no interaction for VISIONxGROUP (*F*(1,54) = 1.52, *p* = 0.224; Fig. 3B).

**Fig. 4:**
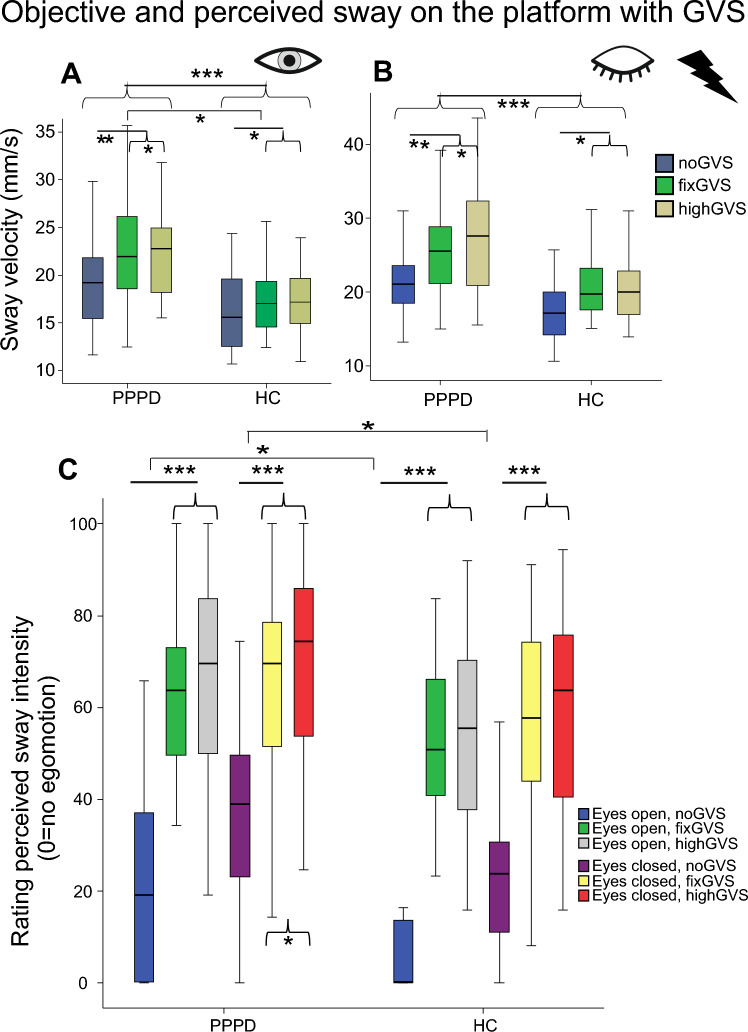
Vestibular stimulation: objective and perceived sway on the firm platform. Postural sway velocity (mm/s) of both groups on the firm platform during GVS with different intensities (fix, high, and noGVS), with the eyes open (**A**) and closed (**B**). The perceived sway intensity on a visual analog scale (0–100, 0 = no egomotion) for the same conditions is shown in (**C**) with the eyes open and closed. Patients show a larger postural instability, irrespective of the visual condition, but there were no group differences in rating of perceived egomotion during effective GVS, i.e., the larger objective sway of the patients is not associated with a stronger egomotion perception when compared with HC. *p = 0.05; **p = 0.01; ***p = 0.001

*Postural sway on the firm platform using GVS*: Using GVS on the firm platform (Fig. 4A) with the eyes open, there were main effects of STIMULATION (*F*(2,55) = 8.63, *p* = 0.001) and GROUP (*F*(1,57) = 12.78, *p* = 0.001) and a trend toward an interaction STIMULATIONxGROUP (*F*(2,56) = 3.28, *p* = 0.050). Post hoc pairwise stimulation comparisons on the firm platform showed significantly larger PSS (i) with GVS (high and fixGVS) compared to noGVS (*p* = 0.015 and *p* = 0.003) and (ii) of patients than HC (Fix and highGVS, both *p* < 0.001). Using GVS with the eyes closed on the firm platform (Fig. 4B), there was a main effect of STIMULATION (*F*(2,56) = 28.83, *p* < 0.001) and for GROUP (*T*(1, 57) = 11.22, *p* = 0.001) but there was no interaction STIMULATIONxGROUP (*F*(2,56) = 0.59, *p* = 0.523). Post hoc pairwise stimulation comparison on the firm platform showed significantly larger PSS (i) with GVS (high and fixGVS) compared to noGVS (both *p* < 0.001) and (ii) of patients compared to HC in each GVS condition (noGVS: *p* = 0.011; fixGVS p = 0.008), highGVS *p* = 0.002).

*Rating of perceived sway on the firm platform applying GVS*: There were main effects of VISION (*F*(1,56) = 42.60, *p* < 0.001), STIMULATION (*F*(2,55) = 153.39, *p* < 0.001) and an interaction VISIONxSTIMULATION (*F*(2,55) = 9.237, *p* = 0.001) but no interaction for VISIONxGROUP, STIMULATIONxGROUP or VISIONxSTIMULATIONxGROUP (*p* > 0.8, *p* > 0.5, Fig. 4C). Pairwise comparison revealed a group difference: patients showed higher ratings of perceived sway (53.4 ± 2.9) than HC (42.5 ± 3.2; *p* = 0.016). This group difference was only significant in the pairwise comparison in the noGVS condition, both with the eyes open and closed. Ratings of fixGVS and highGVS were larger (*p* = 0.001) compared to noGVS (Fig. 4B) with no difference between highGVS and fixGVS.

**Fig. 5:**
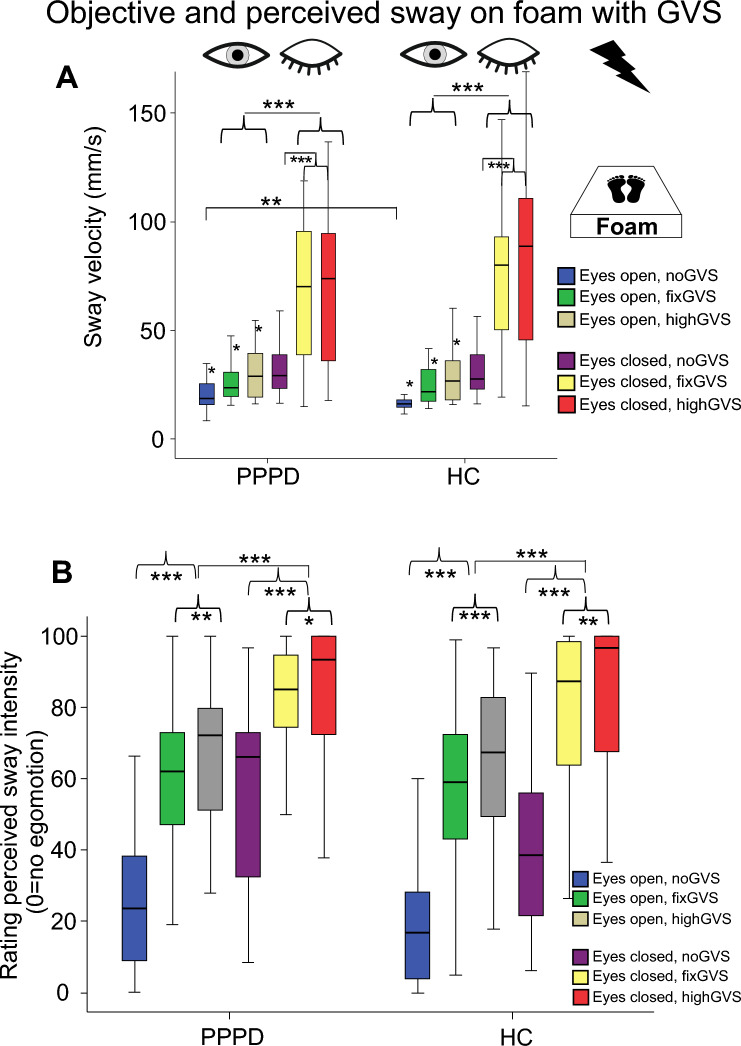
Vestibular stimulation: Objective and perceived sway on foam. Postural sway velocity (mm/s) on foam of both groups during GVS with different intensities (fix, high, and noGVS), with the eyes open and closed (**A**). The perceived sway intensity on a visual analog scale (0–100, 0 = no egomotion) for the same conditions is shown in (**B**) with the eyes open and closed. There are stimulus- and condition-related within-group differences but no group differences. *p = 0.05; **p = 0.01; ***p = 0.001

*Postural sway on foam applying GVS*: there were main effects of VISION (*F*(1,56) = 153.55, p = 0.001) and STIMULATION (F(2,55) = 82.71, *p* < 0.001) but not for GROUP (p = 0.212) (Fig. 5). There was a significant interaction of VISIONxGROUP (*F*(1,56) = 4.67, *p* = 0.03) but not for STIMULATIONxVISION (*F*(2,55) = 3.29, *p* = 0.061) and STIMULATIONxGROUP (*F*(2,55) = 1.99, *p* = 0.159). Post hoc comparisons showed larger PSS of patients with the eyes open in the noGVS condition only (*F*(2,55) = 9.245, *p* = 0.004). PSS in both groups was always larger with the eyes closed and with GVS. Post hoc comparison showed significant PSS differences between all GVS conditions with the eyes open (*p* = always < 0.008) but not between fixGVS and highGVS with the eyes closed.

*Rating of perceived sway during GVS on foam*: There were main effects of VISION (*F*(1,52) = 78.53, *p* < 0.001), STIMULATION (*F*(2,51) = 124.72, *p* < 0.001) and an interaction VISIONxSTIMULATION (*F*(2,51) = 34.84, *p* < 0.001) but there were no interactions for VISIONxGROUP (*p* > 0.15) or STIMULATIONxGROUP (*p* > 0.4) or VISIONxSTIMULATIONxGROUP (*p* > 0.38) (Fig. 5), i.e., participants rated perceived sway higher during eye closure, during GVS with the eyes open and during GVS with the eyes closed but there were no group differences (PPPD: 59.6 ± 3.2; HC 53.8 ± 3.2; *F*(1,52) = 1.666; *p* = 0.202). Pairwise comparison over all visual conditions revealed significant larger sway perceptions during fixGVS (*p* = 0.001) and highGVS (*p* = 0.001) compared to noGVS. HighGVS was rated higher compared to fixGVS (*p* = 0.019). However, using pairwise comparisons with the same visual condition, there were no group differences in rating between different GVS intensities.

### Perceptual-postural ratio

The perceptual-postural ratio (PPR) was calculated by the rating of perceived sway divided by observed sway speed (PSS) for each experimental condition.

**Fig. 6:**
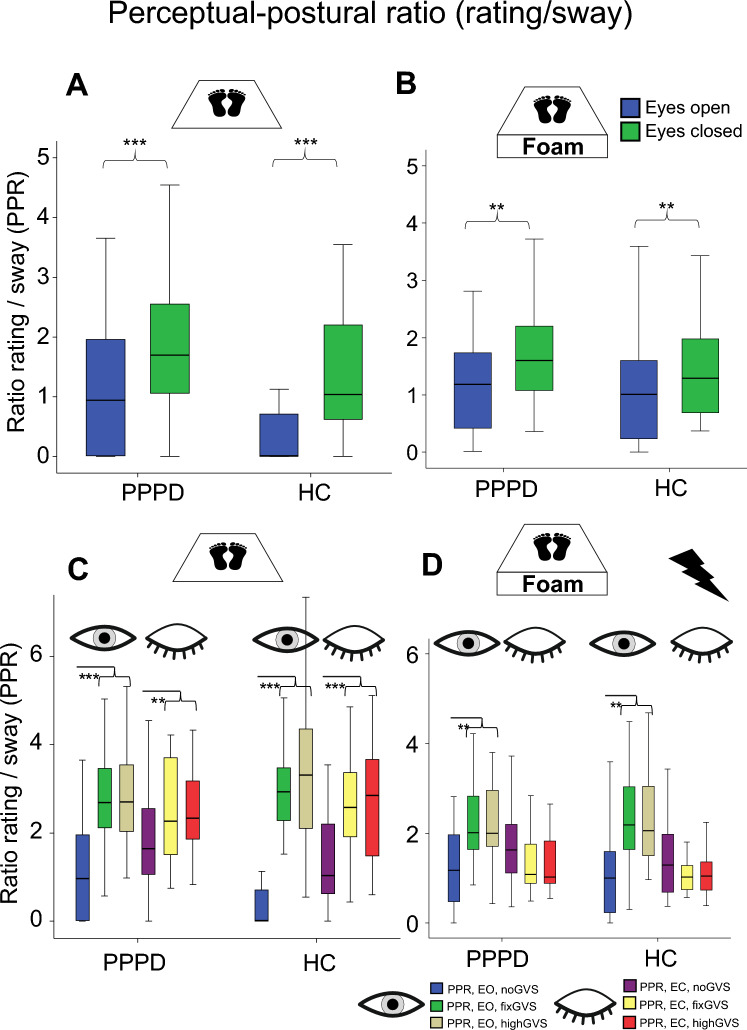
Perceptual-postural ratio (PPR). The ratio of perceived postural instability (PSS) to real sway speed of both groups is displayed for the firm platform (**A**, **C**) and the foam (**B**, **D**) condition with the eyes open and closed, without (**A**, **B**) and with GVS (**C**, **D**). A ratio above one reflects stronger rating of perceived instability compared to real instability. *p = 0.05, **p = 0.01; ***p = 0.001

PPR on the firm platform *without GVS* showed a main effect of VISION (*F*(1,57) = 30.53; *p* < 0.001) with a trend to larger PPR for patients (p = 0.051) but there was no interaction for VISIONxGROUP (Fig. 6A). PPR on foam without GVS showed a main effect of VISION (*F*(1,54) = 6.35; *p* = 0.015) but not of GROUP (p = 0.366) and no interaction VISIONxGROUP (*p* = 0.625, Fig. 6B). Post hoc comparison revealed no group differences.

PPR on the firm platform *with GVS* showed a main effect of STIMULATION (*F*(2,55) = 58.74; *p* < 0.001), but not for VISION (*F*(1,56) = 0.26; *p* = 0.613), and interactions of STIMULATIONxVISION (*F*(2,55) = 34.20; *p* < 0.001) and GROUPxSTIMULATION (*F*(2,55) = 4.47; *p* < 0.027, Fig. 6C). Post hoc between-group comparisons of different GVS intensities did not show significant PPR differences (Fig. 6C). Post hoc within-group comparisons of different GVS intensities showed larger PPR with GVS but no group differences (Fig. 6C).

PPR on the foam with GVS showed main effects of VISION (*F*(1,52) = 51.16; *p* < 0.001) and STIMULATION (*F*(2,51) = 8.74; *p* = 0.002) and an interaction STIMULATION xVISION (*F*(2,51) = 49.41; *p* < 0.001) but not for GROUP (*p* = 0.673) (Fig. 6C) and no interaction for GROUPxSTIMULATION (*F*(2,51 = 0.853; *p* = 0.382). Post hoc between-group comparisons of different GVS intensities did not show significant PPR differences (Fig. 6C). Post hoc within-group comparisons of different GVS intensities only showed significant PPR differences (*p* = always < 0.002) with the eyes open (Fig. 6C).

### Reproduction of perceived sway

**Fig. 7:**
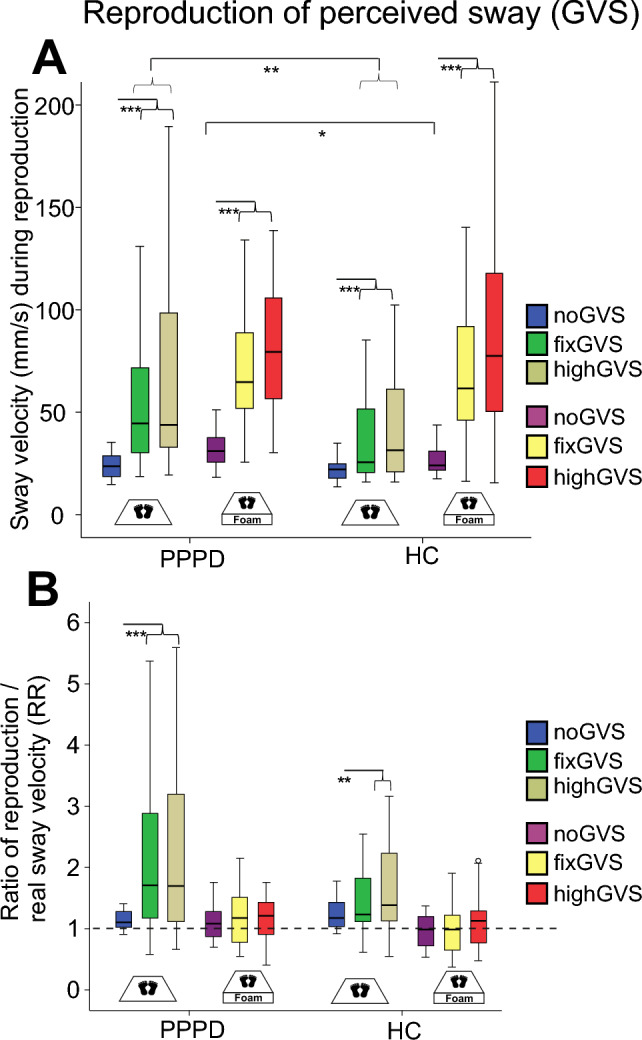
Reproduction of perceived sway during vestibular stimulation (GVS). **A** Postural sway velocity (mm/s) during reproduction of perceived sway. **B** Ratio of sway speed during reproduction/real sway speed (RR) during GVS. The horizontal dashed line indicates when reproduction sway matches postural sway speed during the preceding condition. Box plots are shown for the three GVS stimuli (high, fix, and noGVS) on the firm platform and foam condition separately. *p = 0.05, **p = 0.01; ***p = 0.001

Analyzing sway velocity during reproduction, there were main effects of SOMATOSENSORY (firm platform vs. foam) (*F*(1,57) = 73.98; *p* < 0.001), STIMULATION (*F*(2,56) = 140.34; p < 0.001) and GROUP (*F*(1,57) = 5.06; *p* = 0.028) and an interaction of SOMATOSENSORYxSTIMULATION (firm platform vs. foam, GVS stimulations) (*F*(2,56) = 4.57; *p* = 0.014) and a triple interaction SOMATOSENSORYxSTIMULATIONxGROUP (*F*(2, 56) = 5.60; *p* = 0.006) (Fig. 7).

Calculating the ratio of sway velocity during reproduction/real sway during GVS (reproduction ratio; RR) over all conditions (platform vs. foam, GVS stimulations), there were main effects of STIMULATION (*F*(2,56) = 13.25; *p* < 0.001) and of SOMATOSENSORY (*F*(1,57) = 49.23, *p* < 0.001) but not of GROUP (*p* = 0.274) and no interactions STIMULATIONxGROUP (*p* = 0.341) and SOMATOSENSORYxGROUP (*p* = 0.355). Pairwise comparisons of the same stimulus in the same sensory condition did not reveal significant group differences (Fig. 7). For the firm platform recordings only, there was a main effect of STIMULATION (*F*(2,56) = 14.07; *p* < 0.001) but not of GROUP (*F*(1,56) = 0.23; p = 0.633) and no interaction STIMULATIONxGROUP (*p* = 0.170). Post hoc comparisons of RR on foam revealed a main effect of STIMULATION (*F*(2,56) = 3.39, *p* = 0.043) but neither of GROUP (*p* = 0.175) nor an interaction (STIMULATIONxGROUP, *p* = 0.765).

### Perceptual-postural correlations

The reproduction of perceived sway of the preceding trial with highGVS was predicted in a linear regression analysis by the real sway velocity during highGVS and GROUP. More than 26% of variance was explained by the model (*R*^2^ = 0.518; *F* = (2,57) = 10.26; *p* < 0.001) with a significant beta (= 4.70) of real sway velocity (*T* = 4.10, *p* < 0.001). Reproduction sway velocity of patients increased stronger with sway velocity while standing on the firm platform, both during the noGVS and high GVS (*R*^2^ = 0.268) condition, while it correlated stronger for healthy subjects on foam.

**Fig. 8:**
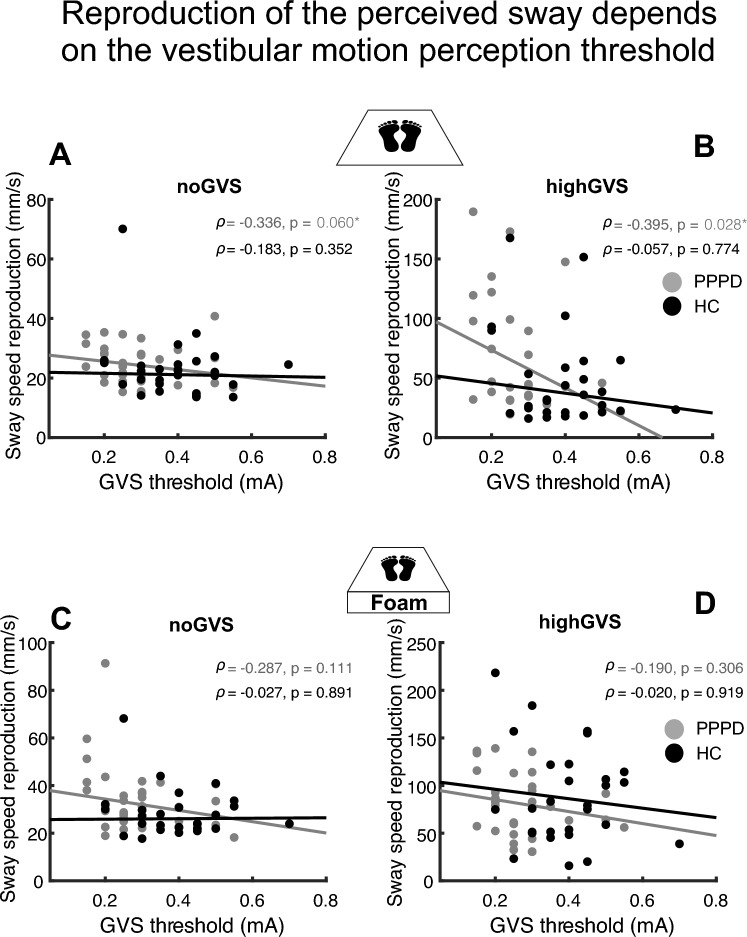
Postural sway speed during reproduction is related to the individual vestibular motion perception threshold (GVS). Postural sway speed during reproduction [of the perceived instability after preceding GVS (**B**) vs. no GVS (**A**)] on the firm platform (**A**, **B**), but not on foam (**C**, **D**), increases with smaller individual vestibular motion perception thresholds of patients (but not of healthy participants). Statistical data in upper right corner are displayed in gray for patients and in black for healthy control subjects

The PSS during reproduction of patients—but not of healthy participants (*p* = always > 0.35)—correlated with the individual thresholds of vestibular (GVS) motion detection in the fixGVS (Spearman-Rho, *ρ* = – 0.473, *p* = 0.007) and highGVS condition (*ρ* = – 0.395; *p* = 0.028) on the firm platform but not on foam (Fig. 8). Accordingly, there was a trend to a negative correlation of the ratio of reproduction (RR) on the firm platform with the individual thresholds of GVS motion detection in the fixGVS and highGVS condition which closely failed the significance level (Spearman-Rho, *ρ* = – 0.321, *p* = 0.078; *ρ* = – 0.355, *p* = 0.050). There was no correlation of PSS during reproduction with the individual GVS threshold in the noGVS condition on the firm platform or with any GVS condition on foam. None of the sway parameters correlated with any of the patients’ clinical scores after correcting for multiple comparisons (disease duration, Niigata, MSSQ, ALQ, NEO-FFI, EQ-5D-3L, HADS, see Table 1).

## Discussion

We investigated postural sway and its perception by vestibular stimulation (GVS) to elucidate whether an abnormal sway perceptual scaling mechanism accounts for the perceived postural instability and egomotion PPPD patients complain during vestibular stimulation (e.g., body movements). We will discuss the posturographic data in the following way: (i) the influence of sensory, specifically vestibular signals on postural stability in PPPD, (ii) the relation of real, perceived and reproduced postural sway, and (iii) the role of individual motion perception thresholds to sensory stimuli for postural influence.

*(i) Influence of sensory, specifically vestibular signals on postural stability in PPPD* Previous posturographic studies with PPPD patients focused on the differential role of somatosensory or visual signals on postural stability by modulating one or several of them at the same time [26]. Symptoms and postural stability of PPPD patients are described to be visually “dependent” [27, 28]. Symptoms exacerbate on exposure to environment with complex stationary or moving visual stimuli [6]. Activity in the visual cortex differs from HC when exposed to moving virtual reality stimuli [14, 15]. Using foam posturography, Romberg’s ratio was larger and foam ratio smaller in PPPD compared to HC, suggesting that visual dependence in PPPD reflects the tendency to rely stronger on visual than on somatosensory inputs for postural control [28]. Unfortunately, conclusions were limited due to the small cohort size and the fact that the majority of patients had persistent vestibulopathy in the study of Ichijo and coworkers. Our patients showed a greater postural imbalance than HC in the absence of visual control on the firm platform but not during attenuated somatosensory feedback (foam). The latter argues against the view of preferred visual dependence of PPPD patients to maintain balance because one would expect poorer postural control under more demanding postural conditions (foam) with the eyes closed. Other studies also did not find weaker postural control during visual deprivation [9, 12, 15, 26, 27] suggesting that visual dependence in PPPD may reflect primarily an increased visual attention or a stronger discomfort with visual control in easily controllable postural condition but does not necessarily reflect poorer balance control mechanisms. Additional interference with concomitant GVS also did not change the larger sway of our patients, independent of visual fixation.

Visual dependence of stationary visual reference targets needs to be separated from the discomfort PPPD patients experience during exposure to a moving visual surrounding where patients showed greater imbalance using complex moving scenes in the Sensory Organization Test [26]. It remains to be tested whether this is related to a stronger visual distractibility or an impaired visual motion perception. We recently found higher visual motion perception thresholds in PPPD, i.e., patients required more coherently moving random dots to perceive a global visual motion than healthy subjects [11]. A poorer complex visual motion recognition, e.g., traffic visual stimuli, may increase anxiety and levels of uncertainty as visuomotor reactions might occur delayed or inappropriate. Alternatively, visual hypersensitivity by complex visual stimulation may provoke a stronger distracting stimulus in PPPD which needs to be tested in the future. Some evidence comes from observations that concomitant large-field moving surroundings by optokinetic stimulation during functional head impulse tests provoked more reading errors on an optotype display on a computer screen in PPPD which was not found without optokinetic stimulation [54].

Weakening somatosensory feedback by foam worsened postural stability indistinguishable in patients and HC, i.e., patients use proprioceptive signals properly for postural control. This was not only found in the baseline foam condition with the eyes closed but also during additional vestibular stimulation (GVS). Vestibular signals did not destabilize posture in PPPD but probably helped to maintain balance. However, when firm somatosensory support is provided, PPPD patients became destabilized with GVS, irrespective of visual control. This seems to be a robust finding as it confirms our previous data in an independent PPPD cohort [9]. The differential impact of additional vestibular stimulation on postural control with vs. without somatosensory feedback should therefore be considered in physiotherapy programs of PPPD patients.

We used two GVS intensities, one with respect to the individual GVS motion perception threshold and one with a fixed perceivable stimulus intensity for better comparability. As both GVS intensities elicited a larger sway of PPPD patients on the firm platform it is unlikely that the larger sway in PPPD is related to their lower individual motion perception threshold. Vestibular motion perception thresholds were not different between groups [11]. The higher sway of PPPD patients during GVS in the least challenging postural conditions (firm platform) may be related to allocation of attention to egomotion perception rather than postural control itself as the imbalance was not found in the dual-task condition [9]. This makes it necessary to address the relation of real and perceived sway.

(ii) *Postural perception: real and perceived postural sway.* When asked to rate the perceived postural instability our PPPD patients showed a larger imbalance on the firm platform and rated this instability greater than HC, i.e., perceived and objective imbalance was proportional. Accordingly, the posturo-perceptual ratio (PPR) did not differ between groups reflecting normal perceptual scaling of the real postural sway. This is in contrast to a related study in which PPPD patients revealed a striking discrepancy with small objective but large perceived instability, reflecting severe postural misperception [29]. In the latter study, postural balance of HC subjects was recorded not on the firm platform (our study) but on foam in the baseline condition to facilitate the perception of postural instability. This may explain why our group comparison of PSS in identical experimental conditions did not reveal postural misperception in patients.

In the more challenging postural condition on foam, patients showed larger sway compared to the firm platform and to HC but rating of perceived sway revealed no group differences potentially implying unproportional posturo-perceptual scaling. However, this was not confirmed by the perceptual-postural ratio which did not reveal significant group differences. Intensity of perceived instability on the firm platform of all participants clearly differed between conditions with vs. without vestibular stimulation, irrespective of visual control, but there were no group differences. Vestibular stimulation during somatosensory (foam) and visual (eyes closed) deprivation markedly increased postural sway but there were no group differences both in real and perceived imbalance. Sway-perceptual scaling in these highly demanding postural control conditions was normal, i.e., not different from HC. According to our data, postural misperception of PPPD patients under stationary postural conditions on the firm platform [29] is not transferable to comparable conditions when additional vestibular stimulation is provided. Vestibular stimulation may even be used to normalize abnormal perceptual-postural scaling and to counteract postural misperception under stationary postural conditions (firm ground).

When asked to reproduce the perceived sway during GVS, patients did not show larger postural sway, neither on the firm platform nor on foam. The reproduction of imbalance due to the perceived egomotion elicited by vestibular stimulation was normal. The perceptual-sway scaling in the reproduction process seems to be intact. The reproduction task not only reflects normal metacognitive judgement of their own posture during GVS (normal posture-perceptual scaling) but also indicates normal perceptual-motor transformation in our patients. Impaired metacognitive performance (judgement about self-performed movements) and reduced confidence of the correctness of visuomotor decisions have been proposed to underlie functional movement disorders [55].

The difference to the related study suggesting postural misperception [29] and possibly impaired metacognitive postural control cannot be explained by the experimental visual conditions as we asked the patients to reproduce perceived postural sway with the eyes open, i.e., the same visual condition. Postural misperception in PPPD with larger reproduced sway may be constrained to the firm platform standing condition [29] as we could neither replicate it, particularly after vestibular stimulation. This should be tested with natural vestibular stimulation in the future (e.g., by accompanying head movements) as it may help to counteract postural misperception by physiotherapy.

(iii) *Role of individual motion perception thresholds for postural influence.* The reproduced postural instability of the preceding sway perception with the eyes closed seems to be related to the individual vestibular motion perception thresholds: the lower the thresholds the higher was the reproduced instability of GVS. This relation was only found during GVS (high, fix) on the firm platform but not on foam. This argues against a low somatosensory dependence of postural control in PPPD [28]. Standing on firm support under visual postural control is the condition in which leg stiffness due to co-contraction of agonist and antagonist muscles occurs in PPPD [23]. This has not been tested on foam but we would expected no co-contraction in this demanding condition.

Given the normal perceptual-postural scaling in the PPPD patients of our study the thresholds of vestibular motion perception deserve further attention. Vestibular chair motion perception thresholds have been described as reduced [10] or not different from HC [11, 12]. Despite having the same threshold of correct egomotion recognition, the threshold of correct responses for rotatory egomotion increased with the number of trials with correct perception in the no-motion condition in patients but not in healthy subjects [11]. Accordingly, the vestibular perception threshold by chair rotation of PPPD patients predicted the probability of making false assignments in the sham condition, i.e., patients who readily recognize the correct egomotion direction were prone to perceive egomotion in the no-motion condition. The role of lower vestibular motion perception thresholds by GVS in this and a previous cohort study [9] becomes evident as they seem to increase the risk of abnormal posturo-perceptual scaling during vestibular stimulation (postural misperception), as the reproduced postural sway (and RR) increased with lower perception thresholds by GVS.

This is only found in the simple standing condition (platform with firm support) which allows attention allocation to egomotion perception. In this condition, the differences between individual motion perception thresholds become evident. Vestibular perception thresholds may therefore be used as a parameter to control the efficacy of vestibular physiotherapy in PPPD patients and should be assessed in the simple standing condition on firm support.

In conclusion, processing of vestibular signals to maintain postural stability seems to be preserved in PPPD, with a normal scaling of postural sway perception. However, the individual vestibular motion perception threshold seems to determine the risk of abnormal scaling of postural perception which is in line with previous findings that vestibular motion perception thresholds of PPPD patients predict the probability of recognizing egomotion in a no-motion condition in PPPD.

## Data Availability

The data are made available upon reasonable request.
